# Local population structure of *Plasmodium*: impact on malaria control and elimination

**DOI:** 10.1186/1475-2875-11-412

**Published:** 2012-12-11

**Authors:** Stella M Chenet, Kristan A Schneider, Leopoldo Villegas, Ananias A Escalante

**Affiliations:** 1 School of Life Sciences, Arizona State University, Tempe, AZ, USA; 2 Center for Evolutionary Medicine and Informatics, The Biodesign Institute, Arizona State University, Tempe, AZ, USA; 3 Department MNI, University of Applied Sciences Mittweida, Mittweida, Germany; 4 Department of Mathematics, University of Vienna, Vienna, Austria; 5 Centro de Investigación de Campo Francesco Vitanza, Tumeremo, Bolívar State, Venezuela; 6 ICF International, International Health & Development Division, Calverton, MD, USA

**Keywords:** Plasmodium, Malaria control, Population structure, Microsatellites, Recrudescence, Linkage disequilibrium

## Abstract

**Background:**

Regardless of the growing interest in detecting population structures in malarial parasites, there have been limited discussions on how to use this concept in control programmes. In such context, the effects of the parasite population structures will depend on interventions’ spatial or temporal scales. This investigation explores the problem of identifying genetic markers, in this case microsatellites, to unveil *Plasmodium *genetic structures that could affect decisions in the context of elimination. The study was performed in a low-transmission area, which offers a good proxy to better understand problems associated with surveillance at the final stages of malaria elimination.

**Methods:**

*Plasmodium vivax* samples collected in Tumeremo, Venezuela, between March 2003 and November 2004 were analysed. Since *Plasmodium falciparum* also circulates in many low endemic areas, *P. falciparum* samples from the same locality and time period were included for comparison. *Plasmodium vivax* samples were assayed for an original set of 25 microsatellites and *P. falciparum* samples were assayed for 12 microsatellites.

**Results:**

Not all microsatellite loci assayed offered reliable local data. A complex temporal-cluster dynamics is found in both *P. vivax *and *P. falciparum*. Such dynamics affect the numbers and the type of microsatellites required for identifying individual parasites or parasite clusters when performing cross-sectional studies. The minimum number of microsatellites required to differentiate circulating *P. vivax *clusters differs from the minimum number of hyper-variable microsatellites required to distinguish individuals within these clusters. Regardless the extended number of microsatellites used in *P. vivax*, it was not possible to separate all individual infections.

**Conclusions:**

Molecular surveillance has great potential; however, it requires preliminary local studies in order to properly interpret the emerging patterns in the context of elimination. Clonal expansions and clusters turnovers need to be taken into account when using molecular markers. Those affect the number and type of microsatellite markers, as well as, the expected genetic patterns in the context of operational investigations. By considering the local dynamics, elimination programmes could cost-effectively use molecular markers. However, population level studies need to consider the local limitations of a given set of loci in terms of providing epidemiologically relevant information.

## Background

There has been a growing interest in the population structure of malarial parasites [1-7]. Population structure (deviation from random mating) is a common phenomenon in nature; it is the result of several processes including inbreeding, epidemic population expansions, and geographic isolation (isolation by distance) [8,9]. In most cases, identifying the population structure of a given infection agent relies on a blind approach that considers “sufficient” gene sampling and a “good” survey of the natural populations [10], where “good” and “sufficient” depend on the underlying question. However, evaluating the importance of genetic structures for control and elimination programmes requires understanding how they provide information with epidemiologic value. As examples, whereas sometimes the population structure is informative *per se* (e.g. gene flow or separating domestic from imported cases); in others it becomes a problem, e.g. clonal expansions may hamper the ability of separating a new infection from a recrudescence case in a drug efficacy study. Thus, population structures affect the interpretation of patterns emerging from molecular markers in contexts that are relevant for control and elimination programmes.

It is worth noting that genetic structures are detectable at the time scales determined by the loci mutation rates. Thus, whereas neutral single nucleotide polymorphisms (SNPs) are widely used for genetic analyses in *Plasmodium falciparum*[11-14] and are currently established in *Plasmodium vivax*[15], their rates of evolution may identify genetic structures that are not informative for elimination programmes in some contexts; e.g. SNPs may not describe the dynamics of haplotypes carrying drug-resistant associated mutations [4]. Those more programmatic applications require markers evolving at mutation rates that can identify events at relatively recent (epidemiologically relevant) time scales.

Microsatellites are useful markers under such circumstances, they allow detecting genetic structures at recent divergence times [16] and they are abundant in the *P. falciparum *and *P. vivax *genomes [17]. In addition, these loci are considered to be selectively neutral, unless they are located near genes under selection (linked), e.g. genes with mutations conferring drug resistance [14,18-20]. These characteristics make them useful in large-scale population genetic studies (including genome scans looking for mutations under selection) and for answering operational relevant questions such as separating new infections from a recrudescent case [5,21-24]. Even though this has been the case in *P. falciparum*, the production of hypnozoites (homologous or heterologous) in *P. vivax *and the high rate of multiple clone infections represent a more complex dynamics which may alter the results of a study. Moreover, regardless their high mutation rate, epidemiologic uses for microsatellite loci should consider the effect of the population structures due to demographic processes such as clonal expansions (clonal genetic structure) in areas with low transmission [2-4,25-28]. Since the population genetic structures are usually unknown *a priori*, this investigation focuses on the importance of pilot studies.

Here, a *P. vivax *population was characterized in a single low transmission area using isolates collected in two consecutive years. Then, the genetic structure in *P. vivax *was ascertained by using 25 microsatellite loci. Although such extended number of loci is rarely used in operational research [1,5], this panel allowed evaluating the variation per locus in the study area and their reproducibility in the laboratory. The data obtained was then used to determine the number and properties of loci required to discriminate different epidemiologic scenarios: identification of individual infections (applicable when trying to separate between recrudescence and new infections) and discrimination between clusters of isolates. In order to better understand the dynamics of genetic structures in *P. vivax*, a sympatric *P. falciparum* population sampled during the same time period was included for comparison. Most studies only consider one of the two species [1-4] limiting the comparisons that can be made in areas where both parasites are endemic. Since these two malarial parasite populations come from a low-transmission area, strong linkage disequilibrium was expected [1-4,27,28]. However, in the case of *P. falciparum*, the population underwent strong drug selection [28]. Thus, if the expansion of a resistant lineage determined the clonal structure [4,28,29] of *P. falciparum*, then it was expected a more stable cluster structure through time in this parasite [4] than the one likely to be observed in *P. vivax *where no drug resistance has been documented*.*

This investigation found that the number and type of microsatellite loci needed for operational research should be tailored given the objective. Indeed, the number of microsatellite loci required to differentiate circulating *P. vivax* clusters differs from the minimum number of hyper-variable microsatellites required to distinguish individuals within these clusters. It was also found that the capacity of separating individual infections with a manageable number of microsatellite loci is limited in low transmission areas as the one under investigation; hence, the use of these loci for separating new infections from recrudescent cases need to take into account such limitations. Both parasites follow similar patterns with alternation of clonal lineages at loci non-liked with drug resistant mutations. Thus, whereas drug resistant haplotypes could be fixed in *P. falciparum*[4,28], cyclical clonal replacements are still taking place. Finally, the inclusion of a temporal sampling scheme in the pilot investigation allowed detecting changes in the genetic structure due to migration or other demographic processes. Overall, this study emphasizes the need to evaluate the local diversity of loci and demography by using a pilot study before designing a molecular epidemiologic investigation.

## Methods

### Study area and *Plasmodium *isolates

Two hundred fifteen blood samples with *Plasmodium *monoinfections (107 with *P. vivax *and 108 infected with *P. falciparum*) were used from a surveillance study in Tumeremo (Bolivar State), Venezuela, between March 2003 and November 2004. Any patient positive for malaria by microscopy was invited to provide a sample for further characterization of the parasite and then treated according to national guidelines. The study protocol was approved by the bioethics commission of the *Instituto de Altos Estudios *Dr. Arnoldo Gabaldon in Venezuela.

Malaria transmission in Venezuela has fluctuated since 2000, and peaked in 2004, 2005 and 2007. In 2004, the number of cases in the country rose to almost 42,000, primarily as a result of an increase in *P. vivax *malaria in Bolivar State. The endemic nature of malaria in the State of Bolivar is determined by migration related to gold mining, particularly on the country’s border with Guyana. Bolivar state has the greatest number of reported cases of malaria (85% of the nationally reported cases), mainly among miners, agricultural workers and indigenous groups [28]. *Plasmodium vivax *is the predominant malaria parasite species in the country followed by *P. falciparum *and *Plasmodium malariae*. *Anopheles darlingi *and *Anopheles marajoara *have been the main vectors identified in the study area [30].

### Microsatellite analysis

Genomic DNA was isolated from whole blood using the QIAamp DNA mini kit (QIAGEN, Valencia, CA). *Plasmodium vivax *samples were assayed for 25 microsatellites [31,32] and *P. falciparum *samples were assayed for 12 microsatellites [33]. Fluorescently labelled PCR products were separated on an Applied Biosystems 3730 capillary sequencer and scored using GeneMarker v1.95 (SoftGenetics LLC). The finding of one or more additional alleles was interpreted as a co-infection with two or more genetically distinct clones in the same isolate (multiple-clone infection, transmitted by one or several mosquitoes) [33]. Additional alleles generating peaks of at least one third the height of the predominant allele were also scored. Missing data (no amplifications) are reported by loci but not considered for defining haplotypes.

### Population genetic analysis

Microsatellite data was formatted using the Microsatellite tool kit [34]. The heterozygosity estimate (*H*_*E*_) was used as a measure of overall genetic diversity. This was defined as $H_{E}=\left[n/\left(n-1\right)\right]\left[1-\sum_{i=1}^{L}p_{i}^{2}\right]$, where n is the number of isolates analysed and p_i_ is the frequency of the i-th allele (i=1, …, L) in the population. *H*_*E*_ gives the average probability that a pair of alleles randomly selected from the population is different. The sampling variance for *H*_*E*_ was calculated as $2\left(n–1\right)/n^{3}\left[2\left(n–2\right)\right]\left[\sum_{i=1}^{L}p_{i}^{3}-{\left(\sum_{i=1}^{L}p_{i}^{2}\right)}^{2}\right]$[35]. To calculate allele frequencies per locus, samples with mixed infections were included. (If a sample contained, e.g., three microsatellite alleles, each was weight by 1/3 to calculate frequencies.) *H*_*E *_was calculated using Mathematica 8 (Wolfram Research, Inc.) (code available on request).

To test whether microsatellite haplotypes clustered as a single geographic population, the model-based clustering algorithm implemented in the Structure 2.1 software was applied [36]. This software uses a Bayesian clustering approach to assign isolates to *K* populations or clusters characterized by a set of allele frequencies at each locus. The number of such populations may be either previously known or unknown. In the context of this investigation, this approach allows for the identification of groups or populations of parasites that could be circulating in this area (Tumeremo, Venezuela). The observed genetic diversity was evaluated at different *K *values (*K *=2 to 10). Given that this clustering algorithm incorporates stochastic simulations, each *K *value was run independently ten times with a burn-in period of 10,000 iterations followed by 50,000 iterations. The admixture model was used in all analysis which allows for the presence of individuals with ancestry in two or more of the *K* populations [36]. Structure harvester v0.6.8 was used to visualize the output from Structure [37]. To facilitate the interpretation of population-genetic clustering results, CLUMPP (Cluster Matching and Permutation Program) was also used. CLUMPP strips away the ‘label switching’ heterogeneity so that the ‘genuine multimodality’ can be detected and quantified [38]. In addition, *distruct *1.1 was used to graphically display the clustering results [39]. The posterior probability for each number of populations or clusters (*K*) is computed and then the *K *value that better explains the genetic data is an estimate of the number of circulating clusters or populations circulating. Whereas such genetic structures are a deviation from the expectations under one population undergoing random mating, each cluster cannot be considered a random mating population on its own since such clusters could represent clonal lineages. Moreover, Fstat 2.9.3.2 [40] was used to calculate F_st _between clusters.

Evidence of linkage disequilibrium between alleles from different loci in parasite populations was analysed with Arlequin 3.11 [41]. A standardized index of association (*I*^S^_A_) was also used to test for evidence of overall multilocus linkage disequilibrium in the Venezuelan population. This test compares the variance (*V*_*D*_) of the number of alleles shared between all pairs of haplotypes observed in the population (*D*) with the variance expected under random association of alleles (*V*_*E*_) as follows: *I*^S^_A _= (*V*_*D *_/ *V*_*E *_− 1) (*r *− 1), where *r *is the number of loci analysed. *V*_*E *_is derived from 10,000 simulated data sets in which alleles were randomly reshuffled among haplotypes. Significant linkage disequilibrium is detected if *V*_*D *_is greater than 95% of the values derived from the reshuffled data sets. Data were analysed with LIAN 3.1 [42]. Pairwise linkage disequilibria (LD) were also calculated. In particular, R [43] was calculated. For each pairwise comparison only those samples were included, in which data was not missing and only one allele occurred at one of the two loci. If multiple alleles for a locus were found in a sample, e.g., four alleles, the respective haplotypes were weighted by ¼to calculate haplotype frequencies. Allele frequencies were calculated as described above (*p*_*i*_) from all included samples. Calculations were implemented in Mathematica 8 (Wolfram Research, Inc.) (code available on request).

Additionally, deviations from pairwise linkage-equilibrium were explored by using Freeman-Halton’s test [44] (generalized Fisher’s exact test for kxm contingency tables) at a significance level of 5%. Of particular note, contingency tables, resulting from pairwise comparison of microsatellite loci, typically have sparse entries, and in many cells the count is less than 5. This renders asymptotic tests (e.g. G-test) inappropriate. Since exact tests require integer-valued cell counts, for each pairwise of microsatellite loci, only samples that had exactly one microsatellite allele at both loci could be included in the analysis. (Therefore, for some pairwise comparisons, fewer samples were included than for calculating R.) As the number of tests performed and size of resulting contingency tables becomes numerically challenging, Monte-Carlo estimates for exact p-values were calculated using 10,000 random iterations. However, for 2x2 tables Fisher’s exact test was always performed. Computations were performed with SAS® (Version 9.2).

### Statistical analysis

To test the hypothesis that the probability of sampling parasites from different clusters differs over time, the Kruskal-Wallis test was performed with the sampling date as the dependent variable and the clusters as the independent variable. The null hypothesis was that the probability of sampling from each cluster was the same during the two years. To test if the chronological sequence of clusters would not appear randomly, the asymptotic test of Barton and David [45] was performed (“generalization” of the Wald-Wolfowitz runs test). In addition, associations of time and cluster-membership were further explored. For this purpose, cluster-membership was arranged as a nominal variable, and sample date - grouped in monthly ranges - as an ordinal variable in a k x l contingency table. The *χ *^2 ^and likelihood-*χ*^2 ^tests were used. The Pearson’s Ø-coefficient, Pearson’s contingency coefficient (*C*_p_), and Cramer’s * V*were also obtained. All statistical tests were performed using SAS® (Version 9.2).

## Results

Twelve *P. falciparum *imperfect microsatellites reported by Anderson *et al.*[33], were tested for their ability to amplify in this population and to demonstrate polymorphism on a set of Venezuelan field isolates from 2003–2004 (Table 1). Suitable markers were considered as those loci that amplified at least 80% of the samples and showed evidence of polymorphism with a clear peak pattern. Microsatellites TA40 and TA87 did not meet the criteria and were not considered in the analysis, but loci TA1, Polyα, TA60, ARA2, Pfg377, PfPK2, TA109, TA81, TA42 and 2490 were included for the population genetic analysis. These markers are distributed across seven chromosomes and no two loci are physically closely linked. Markers TA1 and TA109 are 14.4 cM apart on chromosome 6; markers Pfg377 and PfPk2 are on chromosome 12 and separated by a distance of 45.8 cM; and markers TA81 and TA42 are 68.9 cM apart on chromosome 5. All loci had repeat units of 3bp or more.

**Table 1:** **Characterization of the *P. falciparum *microsatellite loci in all samples analyzed from Venezuela**

**Locus**	**Chr**	**Size range**	**N. of Alleles**	**HE**	**SD**
POLYa	4	160-187	4	0.44	0.06
TA81	5	118-121	2	0.16	0.04
TA42	5	185-202	3	0.59	0.03
TA1	6	169-181	3	0.66	0.02
TA109	6	162-177	3	0.58	0.03
2490	10	81-84	2	0.39	0.04
ARA2	11	62-65	2	0.11	0.04
Pfg377	12	92-95	2	0.31	0.05
PfPK2	12	162-165	3	0.62	0.03
TA60	13	70-79	2	0.43	0.04

For *P. vivax*, a panel of 11 dinucleotide [31] and 14 markers of 3bp or more [32] was tested. A total of 22 microsatellites that consistently amplified the samples and showed polymorphism were selected (Table 2). These markers were distributed in 13 of the 14 chromosomes and linkage disequilibrium has been previously reported between microsatellites MS4-MS5 in chromosome 6 and MS7-MS8 in chromosome 12 [46].

**Table 2:** **Characterization of the *P. vivax *microsatellite loci in all samples analyzed from Venezuela**

**Locus**	**Chr**	Core sequence*	**Size range**	**N. of Alleles**	**%MAF**	**HE**	**SD**
**Dinucleotides**							
2.21	2	AC	92-104	6	7.07	0.73	0.02
4.271	4	AT	86-122	7	9.38	0.30	0.06
6.34	6	AC	138-158	9	7.07	0.79	0.03
7.67	7	AT	98-124	11	13.64	0.76	0.03
8.332	8	AT	216-260	10	6.12	0.77	0.03
10.29	10	AT	110-130	7	5.10	0.69	0.04
12.335	12	AT	156-180	8	6.06	0.71	0.04
14.185	14	AT	266-289	9	5.05	0.61	0.05
**Trinucleotides**							
MS1	3	(GAA)_11_	231-246	6	2.02	0.70	0.03
MS3	4	(GAA)_11_	184-193	4	0	0.54	0.05
MS15	5	(TCT)_10_	236-284	10	2.04	0.77	0.03
MS4	6	(AGT)_18_	189-210	6	3.03	0.53	0.06
MS9	8	(GGA)_18_	155-176	8	8.16	0.77	0.03
MS7	12	(GAA)_9_	145-157	4	4.08	0.52	0.03
**Imperfect**							
MS12	5	(TTC)_10_(TGC)_4_	185-269	9	6.06	0.70	0.04
MS2	6	(TAAA)_2_TATA(TAAA)_6_TATA(TAAA)_19_	181-285	13	12.12	0.82	0.03
MS5	6	CCTCTT(CCT)_11_	172-187	5	5.10	0.58	0.04
MS16	9	(ACA)_9_GCA(ACA)_3_GCA(ACA)_7_GCA(ACA)_3_GCAATC	238-400	14	12.79	0.73	0.04
MS20	10	(GAA)_11_GAG(GAA)_13_(CAA)_4_ GAA(CAA)_5_	193-259	16	19.39	0.80	0.03
MS6	11	(TCC)_2_(TCT)_3_(CCT)_2_(TCC)_2_ GCTTCT(TCC)_10_	211-244	6	2.08	0.74	0.03
MS8	12	(CAG)_2_(CAA)_11_	201-285	14	17.78	0.82	0.02
MS10	13	GAA(GGA)_2_AGA(GGA)_9_AGA(GGA)_4_AGAGGAAGA(GGA_3_)	189-249	12	13.13	0.72	0.03

*Descibed by Imwong et al., 2006 [31] and Karunaweera et al., 2008 [32].

### Multiple infections

In *P. falciparum*, 2.8% of the 108 samples had multiple infections while in *P. vivax* 15.9% out of 107 samples were also found with multiple infections harbouring two or three alleles at two or more loci. When grouping samples according to their date of collection, a greater proportion of mixed clone infections were found in *P. vivax *samples from 2004 (at the peak of malaria transmission in Venezuela), with more than double the amount of mixed infections found in *P. vivax *samples from 2003.

### Genetic diversity

**Figure 1:**
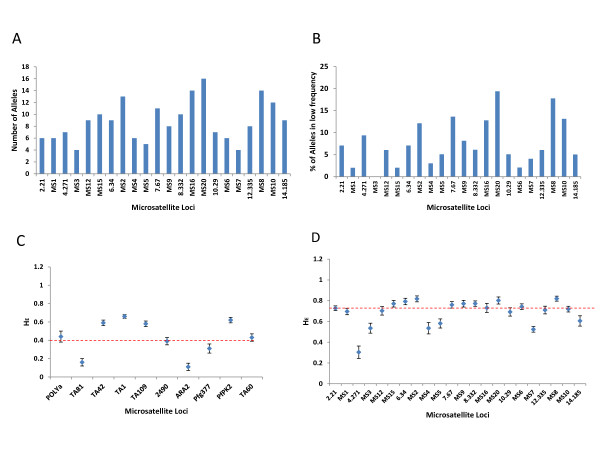
**A) Number of alleles per loci and B) percentage of alleles in low frequency (<5%) in *P. vivax. ***Expected heterozygosity (*H*_*E*_) in **C**) *P. falciparum *and in **D**) *P. vivax *using all samples from Tumeremo.

The frequency distribution of alleles at each locus was determined using all samples; however, three samples each were excluded in *P. falciparum *and *P. vivax *since they only amplified two or none loci. Most of the *P. vivax *loci but MS3, MS7 and MS10 followed (almost) a normal distribution (histogram of microsatellite repeats), which indicates an appropriate sampling of the alleles present in Tumeremo. However, many of the *P. vivax *markers showed a considerable number of (rare) alleles (frequency lower than 5% further referred as Minor Allele Frequency or MAF, see Figure 1 A and B). In *P. falciparum*, the same trend was not observed since only a small number of alleles per loci were found suggesting low genotypic diversity due to recent strong selection by drug pressure and low recombination [28]. Furthermore, note that *P. vivax *relapses from hypnozoites occur, which increases the potential to harbor higher genetic diversity as compared with *P. falciparum *if patients do not comply with the primaquine treatment and as a result it hampers the ability to differentiate between reinfection and recrudesce cases. The expected heterozygosity (*H*_*E*_) was calculated in both species using all microsatellites (Figure 1 C and D). *P.vivax *was found to be highly polymorphic (*H*_*E *_= 0.73 ± 0.032) in comparison to *P. falciparum *(*H*_*E *_= 0.44 ± 0.035). Moreover, the year by year analysis (Additional file 1) revealed higher levels of genetic diversity in samples from 2004 (*H*_*E *_= 0.79 ± 0.0221) than from 2003 (*H*_*E *_= 0.67 ± 0.0301). In *P. falciparum*, the level of genetic diversity in 2003 (*H*_*E *_= 0.51 ± 0.0378) was higher than in 2004 (*H*_*E *_= 0.39 ± 0.0641) and all mixed samples were found during 2003.

### Population structure

**Figure 2:**
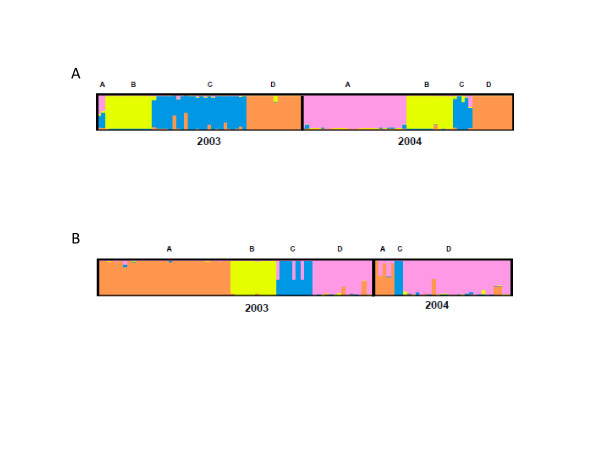
**Population structure of A) *P. falciparum *(cluster A – pink, cluster B – yellow, cluster C – blue and cluster D – orange) and B) *P. vivax *(cluster A – orange, cluster B – yellow, cluster C – blue and cluster D – pink) inferred from microsatellite typing of Tumeremo haplotypes from 2003 and 2004 using the STRUCTURE program.**

The population structures of *P. falciparum *and *P. vivax *were determined using the Structure 2.1 software. Single infections and mixed infections with only one polymorphic locus were included in the analysis with complete data or missing data in no more than three loci. The posterior probability for each cluster (*K*) was computed and the clustering patterns obtained with *K *= 4 was associated with the highest Delta *K *values (Delta *K *= mean (|L”(*K*)|)/sd(L(*K*))) for both species (Figure 2) [37]. Most parasites had their origin clearly assigned to a single cluster; few of them were fairly admixed, showed by bars partitioned into *K *coloured segments. Each fragment in the bar represents the estimated membership fractions, of each isolate or strain, in *K *clusters. However, the algorithm implemented in Structure might detect K-1 reasonable clusters, and subsume remaining low frequency variants in the K^th ^cluster. This might be indicated by higher genetic diversity in just one cluster. In each of the four *P. falciparum *clusters, similar numbers of samples were found (Table 3a); however, in *P. vivax * most of the samples were grouped either in the clusters labeled as “A” or “D” (Table 3b). In addition, when grouping *Plasmodium *haplotypes by year, *P. falciparum *samples from either 2003 or 2004 were also grouped in four clusters. Still, in 2003 haplotypes from falciparum cluster C were overrepresented while in 2004 haplotypes from falciparum cluster A were more abundant. Clusters B and D were present in almost the same frequency during both years. On the other hand, *P. vivax *samples from 2003 did group in four clusters whereas samples from 2004 were grouped in only three clusters; nevertheless, these results might be biased due to the lower number of 2004 samples included in the analysis. Due to the high prevalence of multi-clonal infections (13 out of 41) in the 2004 *P. vivax *samples it was difficult to determine the total number of haplotypes present in the sample so an estimate using the single infections was considered. From this description, it seems that the probability of sampling parasites from different clusters differs over time. The Kruskal-Wallis test was performed and showed to be significant for *P. falciparum *and *P. vivax *(p-values <0.01), indicating that the probabilities of sampling a parasite belonging to a given cluster changes over time. This is an indication of clonal expansions.

**Table 3:** Genetic diversity by clusters

*a).P. falciparum*					
**Cluster**	**Samples**	**Haplotypes**	**N of Alleles**	**HE**	**SD**
A	29	3	1.4	0.12	0.07
B	24	3	1.3	0.07	0.05
C	29	13	2.3	0.38	0.05
D	24	2	1.1	0.01	0.01
*b). P. vivax*					
A	37	15	2.5	0.15	0.04
B	11	7	1.4	0.11	0.04
C	11	4	1.8	0.32	0.05
D	41	37	7.9	0.77	0.03

If parasite prevalence is due to events of clonal expansion, different clusters would be predominant at different time periods. Hence, the chronological sequence of clusters would not appear randomly. Since some samples were taken on the same day, the chronological order on the sample labels was followed. The Barton-David test was significant (p<10^-11 ^for *P. falciparum*, p<10^-7 ^for *P. vivax*) indicating that cluster-membership is not random over time, which is another indication for clonal expansion.

Furthermore, associations of time and cluster-membership were explored. The *X*^2 ^and likelihood-*X*^*2*^-tests were both significant on an alpha level of 5% (all p-values <0.01). For *P. vivax*, *ϕ*  =  0.1790, × *C*_*p*_  =  0.5838, *C*_*p*_  =  0.5838, and *V*  = 0.4151, were obtained, indicating an association between time and cluster-membership. Likewise, for *P. falciparum, ϕ*  = 0.8043, *C*_*p*_  = 0.6267*; *and *V*  = 0.4643 were obtained. However, it should be mentioned that the 85% of the cells in the contingency had expected counts less than 5, which renders the application of *X*^2 ^tests and *X*^2^-based association measures problematic. It should be mentioned that. The sample size was too large to perform Fisher’s exact test, or rather the generalization of Freeman and Halton [44]. The statistics indicate that there is some association between time and cluster membership; however, this association is not very strong. Notably, the association appears stronger in *P. falciparum* than in *P. vivax*, which can be explained by the hypothesis of clonal expansion due to drug resistance. It may be possible that due to relapses in *P. vivax *the pattern of clonal expansion is distorted; however, data on the follow-up primaquine treatments is not available.

By using only six (Polya, TA60, ARA2, Pfg377, TA81 and 2490) of the 10 loci is enough to differentiate the four clusters with a 98% confidence interval. In addition, at least eight loci (TA1, Polya, TA60, Pfg377, PfPK2, TA109, TA42 and 2490) are needed to correctly identify the 41 unique haplotypes found in the 104 *P. falciparum* isolates determined with all ten loci. Thus, the number of loci used does not allow discriminating all individuals.

*Plasmodium vivax *samples were also reanalysed in order to obtain the four clusters and the different identifiable haplotypes from the 94 *P. vivax *samples (100 clones in total within single infections or mixed infections in one locus only). The set of loci used to identify *P. vivax* clusters (four representative loci from the population, which harbours average values of *H*_*E *_and percentage of alleles in low frequency, and two highly polymorphic loci to differentiate maximum number of individuals within cluster) were: locus 2.21, locus 12.335, locus MS1, locus MS6, locus MS8, and locus MS16, whereas the set of loci needed to differentiate the maximum number of haplotypes were: locus 6.34, locus 7.67, locus 8.332, locus MS2, locus MS8, locus MS9, locus MS10, locus MS15, locus MS16 and locus MS20. The F_st _values between clusters in *P. falciparum *and *P. vivax * populations were also calculated to confirm if there was strong genetic differentiation within these subpopulations. The values were significant and ranged between 0.4 to 0.9 in *P. falciparum *and 0.3 to 0.8 in *P. vivax*. These results confirm that there is a strong genetic differentiation within these subpopulations (Table 4).

**Table 4:** **Microsatellite-based genetic differentiation (Fst) between *Plasmodium *clusters (P-value<0.05)**

*a). P.falciparum*			
**Clusters**	A	B	C
B	0.68		
C	0.48	0.67	
D	0.79	0.91	0.66
*b). P.vivax*			
B	0.85		
C	0.79	0.76	
D	0.42	0.36	0.28

### Linkage disequilibrium

**Figure 3:**
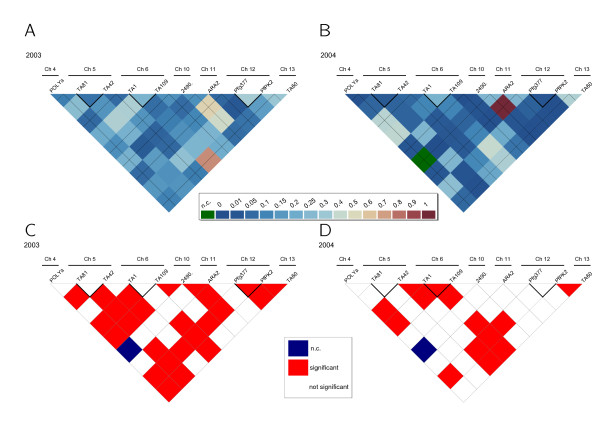
**R values of linkage disequilibrium in *P. falciparum *samples from A) 2003 and B) 2004 and the respective outcomes of Freeman-Halton’s test for significant deviations from linkage equilibrium (C and D) at a significance level of 0.05.**

**Figure 4:**
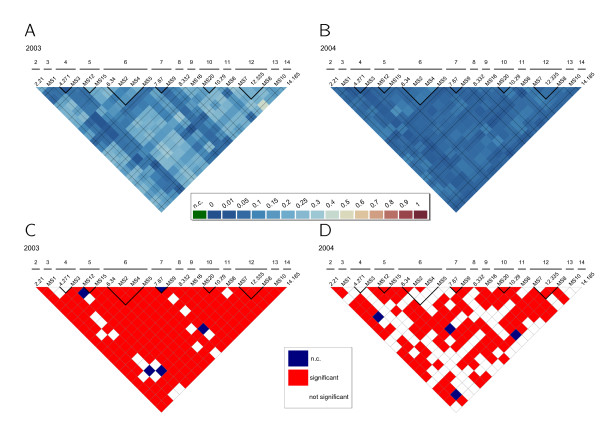
**R values of linkage disequilibrium in *P. vivax *samples from A) 2003 and B) 2004 the respective outcomes of Freeman-Halton’s test for significant deviations from linkage equilibrium (C and D) at a significance level of 0.05.**

In order to calculate linkage disequilibrium, the data was organized to follow the stepwise mutation model when possible. Significant multilocus linkage disequilibrium was found in *P. falciparum *and *P. vivax *isolates according to the standard index of association (I^S^_A2003 _= 0.1236, p<0.01; I^S^_A2004 _= 0.172, p<0.01 and I^S^_A2003 _= 0.3776, p<0.01; I^S^_A2004 _= 0.1448, p<0.01, respectively) and to pairwise comparisons using Arlequin 3.11. Linkage disequilibrium was also calculated considering the time of collection of the isolates, with unique haplotypes and excluding microsatellites in the same chromosome. In all analyses performed significant linkage disequilibrium was found. Further analysis to calculate R in *P. falciparum *(Figures 3, A and B) and *P. vivax *(Figure 4, A and B) conducted between pairs of microsatellites and using all information, including mixed infections, also revealed linkage disequilibrium in both species. Additionally, we tested for pairwise linkage disequilibrium using Freeman-Halton’s (“Fisher’s exact”) test (Figures 3, C and D and Figure 4, C and D). To rule out the possibility that association resulted from combining highly different subpopulations in *P. vivax*, linkage disequilibrium was also calculated using pairwise analysis within the genetic clusters present in both years and a significant result was found for cluster D (I^S^_A_ = 0.1136, p-value <0.001). Furthermore, significant LD (I^S^_A_ = 0.172, p-value <0.05) was also found in *P. vivax *cluster B, which was only present in 2003.

## Discussion

Low-transmission areas are of great interest because they are potential targets for malaria elimination. Even further, they allow for testing how to deploy resources effectively at the final stages of the elimination process. Under such conditions, the precise monitoring of malaria infections is indispensable. Whereas molecular marker-based approaches could enrich malaria surveillance [5], it needs to be ascertained whether such methods - besides their general popularity - are cost-effective for detecting substructures in parasite populations that are valuable for elimination programmes, especially in these low-transmission settings. One step in the process of translating population-genetic concepts into epidemiology is to determine whether an available set of markers has the sensitivity to detect patterns that are epidemiologically informative, especially when performing cross-sectional studies in a narrow period of time that simply allow to see a predominant clonal expansion. Whereas, hypothetically, the number of markers could be increased up to levels that can rule out many confounding factors; in reality, malaria elimination programmes face limited funds, samples, and human resources that can be invested in molecular surveillance.

In general, highly polymorphic loci are suitable to identify individuals within populations whereas more conserved (less polymorphic) loci are useful to establish clusters or parasite populations within a given endemic area. For the first purpose, imperfect microsatellites would be more appropriate due to the complexity on their core sequence which results in greater allelic diversity, while dinucleotides and trinucleotides are more conserved and best represent the local population diversity. However, there are always exceptions in each of the categories established. Distinctions between loci have practical consequences. Highly polymorphic markers have many alleles in low frequency that are more likely to fluctuate by random events (e.g. random genetic drift but also inadequate sampling); thus, a local genotype could not be detected at a given time and then “appear” as new (e.g. been identified as an introduction) after deploying control interventions. Those cases could be considered “imported” rather than the result of residual transmission, misleading the programme to conclude that the control strategy was more efficacious than in reality. This problem is particularly important if the only baseline information is provided by a cross-sectional study that simply detected a predominant clonal expansion. On the contrary, the number of treatment “failures” (putative recrudescent cases) could be over-estimated and conclude that a drug treatment has reduced its efficacy by using low polymorphic marker that cannot properly differentiate genotypes within a cluster. Thus, in order to get a fine scale fingerprint of the epidemiologically relevant events targeted by a given molecular surveillance programme, the number and kind of microsatellites used should consider the characteristics of the population under study. Those characteristics include the replacement of clusters at least between two transmission seasons.

**Figure 5:**
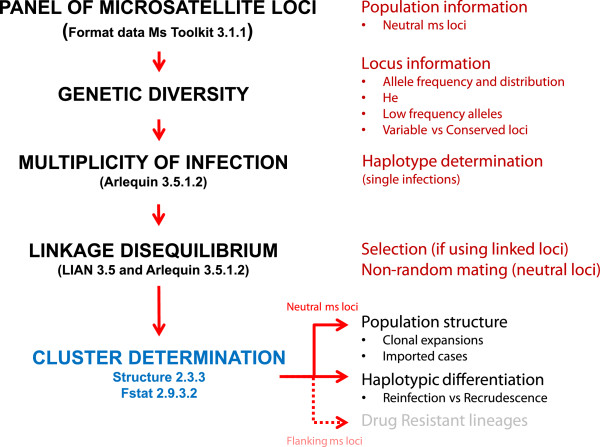
**Flow chart illustrating the steps to follow in a *Plasmodium *population genetic study. **The goal is to identify appropriate microsatellite markers for haplotypic determination (to differentiate between reinfection and recrudescence cases) and to determine clusters or subpopulations (to evaluate clonal expansions and possible imported cases).

This investigation provides some basic information about how to design a pilot (baseline) investigation that could support prospective molecular population-based studies, especially in areas with low genetic diversity and low transmission as the one found in Tumeremo, Venezuela. Those steps are summarized in a flow chart (Figure 5). First, an extended set of loci should be evaluated in order to determine how many reliable work (e.g. can be consistently amplified or the pattern in the electropherogram is unambiguously interpretable) in that area. Then, based on that set of loci, the number of mixed infections, haplotypes and clusters circulating in a given geographic area during at least two transmission seasons can be evaluated. The assumption is that the information collected from the original set of loci (in this case, 22 loci in *P. vivax*) provides the best possible resolution given the available resources. Based on the identified structures using the extended set of loci, it is possible to estimate the minimum number of microsatellite loci needed to detect such structure and differentiate *Plasmodium* populations (clusters) in that specific geographic area. The same baseline data can be used to estimate the minimum number of microsatellites needed to differentiate individuals within these clusters. Hypothetically, since each of the samples analysed originated from a distinct individual, many confounding effects could be reduced by simply increasing the number of markers. However, the number of loci that can be used in a given study is finite, simply because they are tied to the available resources. Thus, pilot studies are needed in order to determine the best resolution that can be obtained using molecular markers in a given context.

Considering all data analysed for *P. falciparum *and *P. vivax*, only 45% of the *P. falciparum *and 85% of the *P. vivax *samples at the individual level were correctly identified. In order to increase the percentage of individual identification for *P. falciparum *in a place like Tumeremo that underwent a strong drug selective pressure, a greater number of polymorphic loci are needed since the heterozygosity per locus is much lower compared with *P. vivax*. This represents a problem since the analysis of more than 10 loci per sample could be costly. It is also important to emphasize that there are clusters of individuals infected by highly related parasites (clonal expansions) and those simply cannot be easily separated. Thus, a manageable number of microsatellites will only identify haplotypes or clonal lineages per population, some of them stable through time [4,47,48]. Population-based studies should be designed considering such restrictions by targeting groups (e.g. arms in a drug efficacy study) or geographic areas where the turnover of such clones (or their persistence) could be informative at the population level. In the case of drug efficacy studies, the proportions of undifferentiated genotypes from prior and post treatment samples can be compared between groups providing a way to reduce the confounding effect of the local parasite demography. Unfortunately, such an approach may require high sample sizes.

*Plasmodium vivax* and *P. falciparum* circulating in Tumeremo differ according to the prevalence of mixed clone infections and the overall levels of genetic diversity. Since in *P. falciparum* drug resistant haplotypes are fixed [4,28], it was expected that such early drug pressure will result in few clones present year-round [4]. However, both species exhibited significant linkage disequilibrium (LD), similar number of clusters/clones when considering haplotypes only, and a similar pattern of temporal replacement of clusters. In *P. falciparum *the four clusters found in 2003 were also found the following year. In *P. vivax*, however, three of the four clusters persisted through time. This could indicate a sporadic event in 2003 of a parasite lineage (cluster B) coming into the Tumeremo population from one of the shared borders with Guyana and Brazil. Moreover, significant LD was found in *P. vivax *within cluster B, which strengthens the idea of a group of minor frequency lineages that are not stable in time and that could be the result of imported cases. Detecting introductions is important especially in the context of control and elimination of the disease; those migration events could explain a local increase in incidence indicating that the deployed strategies/policies were effective in controlling the previously circulating parasites. Ideally, data from other populations should allow identifying the source of the migrant cases providing additional information about how malaria is maintained at a regional level.

A higher level of LD in *P. vivax *compared to *P. falciparum* was not expected since this could not be accounted by recent epidemic expansions of drug resistant lineages like in *P. falciparum*[4,28]. Thus, the pattern observed in *P. vivax *is indicative of ongoing reduced levels of recombination due to the parasite demography. Overall, the local ecology appears to explain the turnover of clones/clusters in both parasites at this time scale. The temporal interval used in this study is appropriate in the context of surveillance when monitoring malaria prevalence from one malaria season to the next one. Plus, the effectiveness of local malaria control can be evaluated by observing changes (which could be the result of incoming gene flow) in the parasite population structure.

It is worth noting that in *P. vivax*, also cluster D had a significant level of LD among lineages within that cluster. When explored closely, this cluster D included different lineages found in low frequency that are stable through time. This cluster D then contrasts with cluster B; where the first seems to be stable in the population and composed by several minor haplotypes, the later (B) may indicate migration/introduction of haplotypes in lower frequency into the endemic area. Thus, a close observation of the linkage disequilibrium within clusters provides additional information that could explain the observed patterns of malaria transmission.

## Conclusions

Molecular epidemiological studies should consider temporal heterogeneities in the parasite population structures. In the context of this investigation, clonal expansions in South America affect estimates of the local genetic diversity. Such dynamic will generate a distorted view if studies are based in a sample collected in a single time point. A second observation is that, with a reasonable number of loci, there is uncertainty in the separation of infections at individual level. Such uncertainties need to be incorporated whenever recrudescent cases need to be separated from new infections in the context of drug efficacy studies. Third, it is relatively easy to identify major clones or sub-populations with a small number of polymorphic microsatellites. Such information could be valuable to better understand gene flow and patterns of migration/re-introduction. It seems logical to contrast samples from at least two transmission seasons, even in gene flow studies, and use loci that allow detecting spatial connectivity so they are useful to track reintroductions at a local level or spatial movements at a regional level. Finally, it was observed that both *P. falciparum *and *P. vivax *exhibit similar patterns of clonal expansions. Whereas previous studies have found strong linkage disequilibrium in both parasites [1-5,7], this is the first time that such dynamics are described in sympatric populations through time. Overall, it seems that such clonal temporal replacements take place even when drug resistant mutations could be fixed, as is the case for *P. falciparum*[4,28].

The type of pilot study proposed in this article could be performed in any endemic area but it is particularly critical in those where clonal expansions are suspected, such is the case of seasonal and low transmission endemic areas. Even though only a limited number of loci could be considered for routine analysis, the use of well-chosen microsatellite loci represent a high sensitive method for cluster differentiation and provides a way to deeper analyse patterns of gene flow as well as parasite lineages maintained through time. Overall, this investigation highlights the need of locally evaluating the diversity of microsatellite loci during at least two transmission seasons before starting molecular epidemiologic investigations.

## Competing interests

The authors declare that they have no competing interests.

## Authors' contributions

SC conducted the molecular genetic studies, performed the genetic diversity experiments, analysed the data and wrote the first draft of the manuscript. KS performed the statistical analysis and contributed to the write up of the final draft. LV provided the samples and contributed to the editing of the manuscript. AE designed the project, supervised and directed the research and contributed to the writing and editing of the manuscript. All authors read and approved the final manuscript.

## Supplementary Material

Additional file 1 **Number of alleles per loci and expected heterozygosity per year in *P. vivax *(A and B) and in *P. falciparum *(C and D) using all samples from Tumeremo.** Click here for file

## References

[B1] Imwong M Nair S Pukrittayakamee S Sudimack D Williams JT Mayxay M Newton PN Kim JR Nandy A Osorio L Carlton JM White NJ Day NP Anderson TJ Contrasting genetic structure in Plasmodium vivax populations from Asia and South America Int J Parasitol 2007 37 1013 1022 10.1016/j.ijpara.2007.02.010 17442318

[B2] Van den Eede P Van der Auwera G Delgado C Huyse T Soto-Calle VE Gamboa D Grande T Rodriguez H Llanos A Anné J Erhart A D'Alessandro U Multilocus genotyping reveals high heterogeneity and strong local population structure of the Plasmodium vivax population in the Peruvian Amazon Malar J 2010 9 151 10.1186/1475-2875-9-151 20525233 PMC2898784

[B3] Pumpaibool T Arnathau C Durand P Kanchanakhan N Siripoon N Suegorn A Sitthi-Amorn C Renaud F Harnyuttanakorn P Genetic diversity and population structure of Plasmodium falciparum in Thailand, a low transmission country Malar J 2009 8 155 10.1186/1475-2875-8-155 19602241 PMC2722663

[B4] Griffing SM Mixson-Hayden T Sridaran S Alam MT McCollum AM Cabezas C Marquiño Quezada W Barnwell JW De Oliveira AM Lucas C Arrospide N Escalante AA Bacon DJ Udhayakumar V South American Plasmodium falciparum after the malaria eradication era: clonal population expansion and survival of the fittest hybrids PLoS One 2011 6 e23486 10.1371/journal.pone.0023486 21949680 PMC3174945

[B5] Arnott A Barry AE Reeder JC Understanding the population genetics of Plasmodium vivax is essential for malaria control and elimination Malar J 2012 10 11 10.1186/1475-2875-11-14 PMC3298510 22233585

[B6] Chenet SM Tapia LL Escalante AA Durand S Lucas C Bacon DJ Genetic diversity and population structure of genes encoding vaccine candidate antigens of Plasmodium vivax Malar J 2012 11 68 10.1186/1475-2875-11-68 22417572 PMC3330009

[B7] Iwagami M Fukumoto M Hwang SY Kim SH Kho WG Kano S Population structure and transmission dynamics of Plasmodium vivax in the Republic of Korea based on microsatellite DNA analysis PLoS Negl Trop Dis 2012 6 e1592 10.1371/journal.pntd.0001592 22509416 PMC3317904

[B8] Wright S Isolation by distance Genetics 1943 2 114 38 17247074 10.1093/genetics/28.2.114 PMC1209196

[B9] Meirmans PG The trouble with isolation by distance Mol Ecol 2012 21 2839 46 10.1111/j.1365-294X.2012.05578.x 22574758

[B10] Gauthier C Tibayrenc M Population structure of malaria parasites: the driving epidemiological forces Acta Trop 2005 3 241 250 15840463 10.1016/j.actatropica.2005.04.001

[B11] Mu J Awadalla P Duan J McGee KM Joy DA McVean GA Su XZ Recombination hotspots and population structure in Plasmodium falciparum PLoS Biol 2005 3 e335 10.1371/journal.pbio.0030335 16144426 PMC1201364

[B12] Volkman SK Sabeti PC DeCaprio D Neafsey DE Schaffner SF Milner DA Jr Daily JP Sarr O Ndiaye D Ndir O Mboup S Duraisingh MT Lukens A Derr A Stange-Thomann N Waggoner S Onofrio R Ziaugra L Mauceli E Gnerre S Jaffe DB Zainoun J Wiegand RC Birren BW Hartl DL Galagan JE Lander ES Wirth DF A genome-wide map of diversity in Plasmodium falciparum Nat Genet 2007 39 113 119 10.1038/ng1930 17159979

[B13] Neafsey DE Schaffner SF Volkman SK Park D Montgomery P Milner DA Jr Lukens A Rosen D Daniels R Houde N Cortese JF Tyndall E Gates C Stange-Thomann N Sarr O Ndiaye D Ndir O Mboup S Ferreira MU Moraes Sdo L Dash AP Chitnis CE Wiegand RC Hartl DL Birren BW Lander ES Sabeti PC Wirth DF Genome-wide SNP genotyping highlights the role of natural selection in Plasmodium falciparum population divergence Genome Biol 2008 9 R171 10.1186/gb-2008-9-12-r171 19077304 PMC2646275

[B14] Cheeseman IH Miller BA Nair S Nkhoma S Tan A Tan JC Al Saai S Phyo AP Moo CL Lwin KM McGready R Ashley E Imwong M Stepniewska K Yi P Dondorp AM Mayxay M Newton PN White NJ Nosten F Ferdig MT Anderson TJ A major genome region underlying artemisinin resistance in malaria Science 2012 336 79 82 10.1126/science.1215966 22491853 PMC3355473

[B15] Orjuela-Sánchez P Karunaweera ND da Silva-Nunes M da Silva NS Scopel KK Gonçalves RM Amaratunga C Sá JM Socheat D Fairhust RM Gunawardena S Thavakodirasah T Galapaththy GL Abeysinghe R Kawamoto F Wirth DF Ferreira MU Single-nucleotide polymorphism, linkage disequilibrium and geographic structure in the malaria parasite Plasmodium vivax: prospects for genome-wide association studies BMC Genet 2010 11 65 20626846 10.1186/1471-2156-11-65 PMC2910014

[B16] Payseur BA Jing P Haasl RJ A genomic portrait of human microsatellite variation Mol Biol Evol 2011 28 303 312 10.1093/molbev/msq198 20675409 PMC3002246

[B17] Russell B Suwanarusk R Lek-Uthai U Plasmodium vivax genetic diversity: microsatellite length matters Trends Parasitol 2006 22 399 401 10.1016/j.pt.2006.06.013 16837246

[B18] Wootton JC Feng X Ferdig MT Cooper RA Mu J Baruch DI Magill AJ Su XZ Genetic diversity and chloroquine selective sweeps in Plasmodium falciparum Nature 2002 418 320 323 10.1038/nature00813 12124623

[B19] McCollum AM Basco LK Tahar R Udhayakumar V Escalante AA Hitchhiking and selective sweeps of Plasmodium falciparum sulfadoxine and pyrimethamine resistance alleles in a population from central Africa Antimicrob Agents Chemother 2008 52 4089 4097 10.1128/AAC.00623-08 18765692 PMC2573158

[B20] McCollum AM Schneider KA Griffing SM Zhou Z Kariuki S Ter-Kuile F Shi YP Slutsker L Lal AA Udhayakumar V Escalante AA Differences in selective pressure on dhps and dhfr drug resistant mutations in western Kenya Malar J 2012 11 77 10.1186/1475-2875-11-77 22439637 PMC3338400

[B21] Nyachieo A VAN Overmeir C Laurent T Dujardin JC D'Alessandro U Plasmodium falciparum genotyping by microsatellites as a method to distinguish between recrudescent and new infections AmJTrop Med Hyg 2005 73 210 3 16014861

[B22] Greenhouse B Myrick A Dokomajilar C Woo JM Carlson EJ Rosenthal PJ Dorsey G Validation of microsatellite markers for use in genotyping polyclonal Plasmodium falciparum infections AmJTrop Med Hyg 2006 75 836 842 PMC1697796 17123974

[B23] Orjuela-Sánchez P da Silva NS da Silva-Nunes M Ferreira MU Recurrent parasitemias and population dynamics of Plasmodium vivax polymorphisms in rural Amazonia AmJTrop Med Hyg 2009 81 961 968 10.4269/ajtmh.2009.09-0337 19996423

[B24] Restrepo E Imwong M Rojas W Carmona-Fonseca J Maestre A High genetic polymorphism of relapsing P. vivax isolates in northwest Colombia Acta Trop 2011 119 23 29 10.1016/j.actatropica.2011.03.012 21497586 PMC3485554

[B25] Sunnucks P Efficient genetic markers for population biology Trends Ecol Evol 2000 15 199 203 10.1016/S0169-5347(00)01825-5 10782134

[B26] Mwangi JM Omar SA Ranford-Cartwright LC Comparison of microsatellite and antigen-coding loci for differentiating recrudescing Plasmodium falciparum infections from reinfections in Kenya Int J Parasitol 2006 36 329 336 10.1016/j.ijpara.2005.10.013 16442537

[B27] Schneider KA Kim Y An analytical model for genetic hitchhiking in the evolution of antimalarial drug resistance Theor Popul Biol 2010 78 93 108 10.1016/j.tpb.2010.06.005 20600206 PMC2916054

[B28] McCollum AM Mueller K Villegas L Udhayakumar V Escalante AA Common origin and fixation of Plasmodium falciparum dhfr and dhps mutations associated with sulfadoxine-pyrimethamine resistance in a low-transmission area in South America Antimicrob Agents Chemother 2007 51 2085 2091 10.1128/AAC.01228-06 17283199 PMC1891388

[B29] Schneider KA Kim Y Approximations for the hitchhiking effect caused by the evolution of antimalarial-drug resistance J Math Biol 2011 62 789 832 10.1007/s00285-010-0353-9 20623287 PMC3242009

[B30] Moreno JE Rubio-Palis Y Páez E Pérez E Sánchez V Abundance, biting behaviour and parous rate of anopheline mosquito species in relation to malaria incidence in gold-mining areas of southern Venezuela Med Vet Entomol 2007 21 339 349 10.1111/j.1365-2915.2007.00704.x 18092972

[B31] Imwong M Sudimack D Pukrittayakamee S Osorio L Carlton JM Day NP White NJ Anderson TJ Microsatellite variation, repeat array length, and population history of Plasmodium vivax Mol Biol Evol 2006 23 1016 1018 10.1093/molbev/msj116 16507919

[B32] Karunaweera ND Ferreira MU Munasinghe A Barnwell JW Collins WE King CL Kawamoto F Hartl DL Wirth DF Extensive microsatellite diversity in the human malaria parasite Plasmodium vivax Gene 2008 410 105 112 10.1016/j.gene.2007.11.022 18226474

[B33] Anderson TJ Su XZ Bockarie M Lagog M Day KP Twelve microsatellite markers for characterization of Plasmodium falciparum from finger-prick blood samples Parasitology 1999 119 113 125 10.1017/S0031182099004552 10466118

[B34] Park SDE Trypanotolerance in West African Cattle and the Population Genetic Effects of Selection. PhD thesis 2001 University of Dublin Microsatellite tool kit version 3.1.1 available from: http://www.animalgenomics.ucd.ie/sdepark/ms-toolkit/

[B35] Nash D Nair S Mayxay M Newton PN Guthmann JP Nosten F Anderson TJ Selection strength and hitchhiking around two anti-malarial resistance genes Proc Biol Sci 2005 272 1153 1161 10.1098/rspb.2004.3026 16024377 PMC1559806

[B36] Pritchard JK Stephens M Donnelly P Inference of population structure using multilocus genotype data Genetics 2000 155 945 959 Structure 2.3.3 available from: http://pritch.bsd.uchicago.edu/structure.html 10835412 10.1093/genetics/155.2.945 PMC1461096

[B37] Dent Earl A vonHoldt Bridgett M STRUCTURE HARVESTER: a website and program for visualizing STRUCTURE output and implementing the Evanno method Conserv Genet Resour 2012 4 359 361 Structure harvester available from: http://taylor0.biology.ucla.edu/struct_harvest/10.1007/s12686-011-9548-7

[B38] Jakobsson M Rosenberg NA CLUMPP: a cluster matching and permutation program for dealing with label switching and multimodality in analysis of population structure Bioinformatics 2007 23 1801 1806 CLUMPP 1.1.2 available from: http://www.stanford.edu/group/rosenberglab/clumpp.html 10.1093/bioinformatics/btm233 17485429

[B39] Rosenberg NA Distruct: a program for the graphical display of population structure Molecular Ecology Notes 2004 4 137 138 Distruct 1.1 available at: http://www.stanford.edu/group/rosenberglab/distruct.html

[B40] Goudet J FSTAT Version 1.2: a computer program to calculate F-statistics J. Heredity 1995 86 485 486 FSTAT 2.9.3.2 available from: http://www2.unil.ch/popgen/softwares/fstat.htm

[B41] Laval EG Schneider S Arlequin (version 3.0): an integrated software package for population genetics data analysis Evol Bioinform 2005 1 47 50 PMC2658868 19325852

[B42] Haubold B Hudson RR LIAN 3.0: detecting linkage disequilibrium in multilocus data Bioinformatics 2000 16 847 848 LIAN 3.5 available from: http://adenine.biz.fh-weihenstephan.de/cgi-bin/lian/lian.cgi.pl. 10.1093/bioinformatics/16.9.847 11108709

[B43] Maruyama T Kimura M Stochastic integrals and their application to population genetics Molecular Evolution, Protein Polymorphism and the Neutral Theory 1982 Tokyo: Japan Scientific Societies Press 151 166

[B44] Freeman GH Halton JH Note on an Exact Treatment of Contingency, Goodness of Fit and Other Problems of Significance Biometrika 1951 38 141 149 14848119

[B45] Barton DE David F Multiple runs Biometrika 1957 44 168 170

[B46] Ferreira MU Karunaweera ND da Silva-Nunes M da Silva NS Wirth DF Hartl DL Population structure and transmission dynamics of Plasmodium vivax in rural Amazonia J Infect Dis 2007 195 1218 1226 10.1086/512685 17357061

[B47] Urdaneta L Lal A Barnabe C Oury B Goldman I Ayala FJ Tibayrenc M Evidence for clonal propagation in natural isolates of Plasmodium falciparum from Venezuela Proc Natl Acad Sci USA 2001 98 6725 6729 10.1073/pnas.111144998 11371616 PMC34420

[B48] Razakandrainibe FG Durand P Koella JC De Meeüs T Rousset F Ayala FJ Renaud F "Clonal" population structure of the malaria agent Plasmodium falciparum in high-infection regions Proc Natl Acad Sci USA 2005 102 17388 93 10.1073/pnas.0508871102 16301534 PMC1297693

