# Supplementary figures and images for: Local population structure of Plasmodium: impact on malaria control and elimination
Source: Malar J. 2012 Dec 11;11:412. doi: 10.1186/1475-2875-11-412 (PMC3538601; doi:10.1186/1475-2875-11-412)

**A**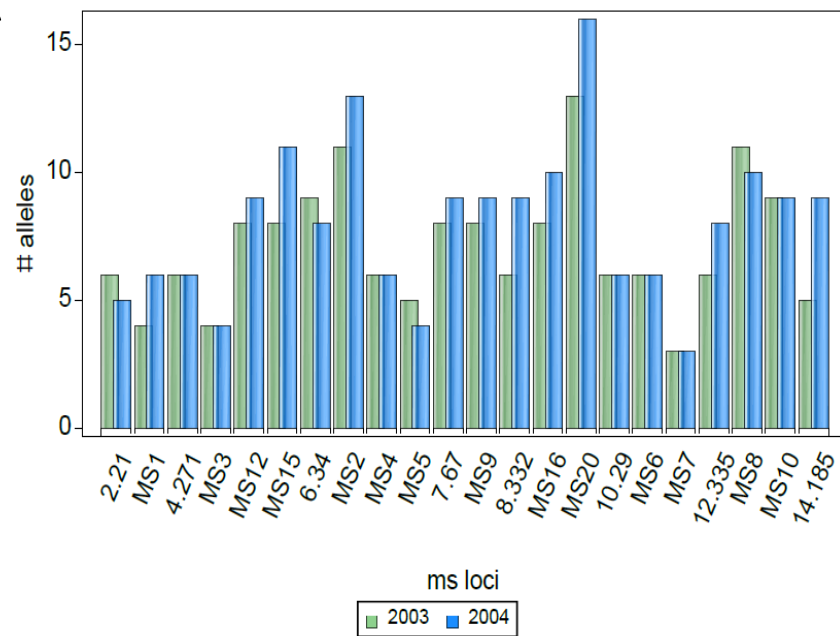**B**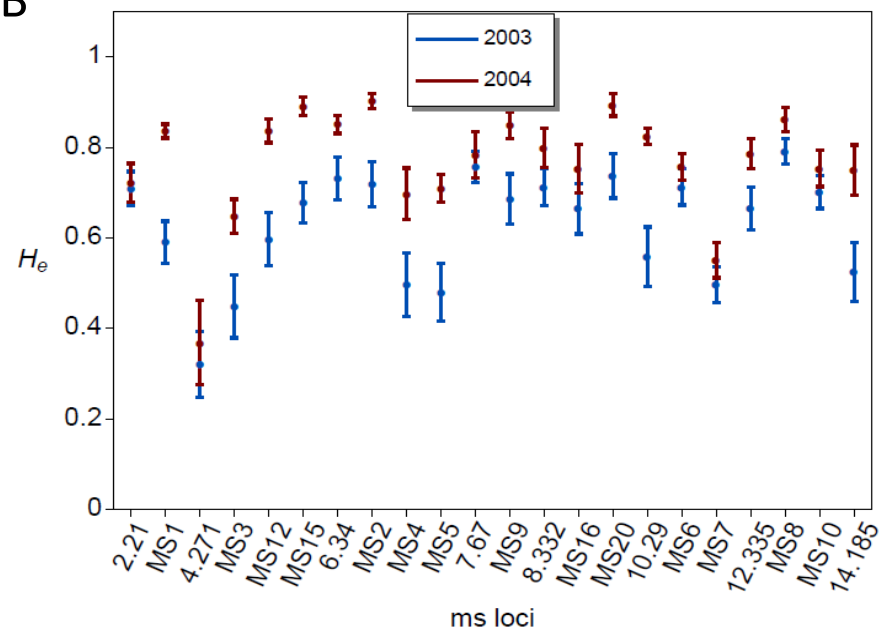**C**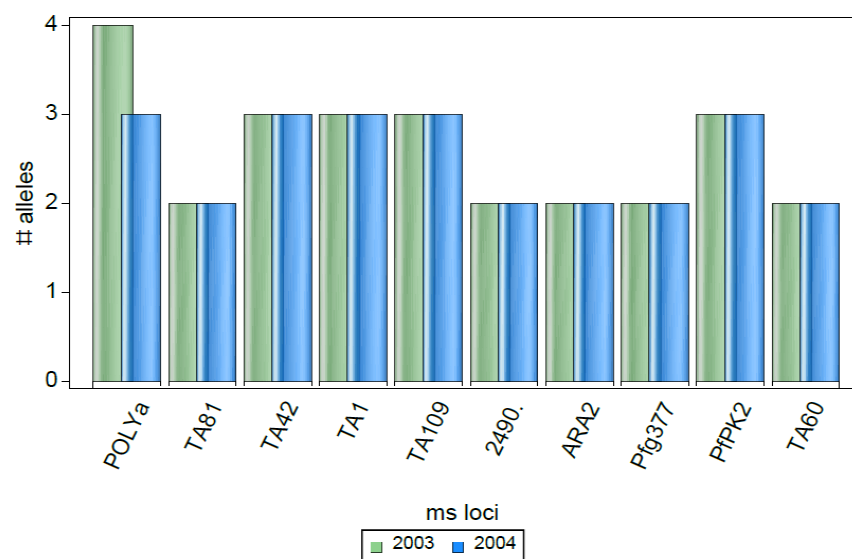**D**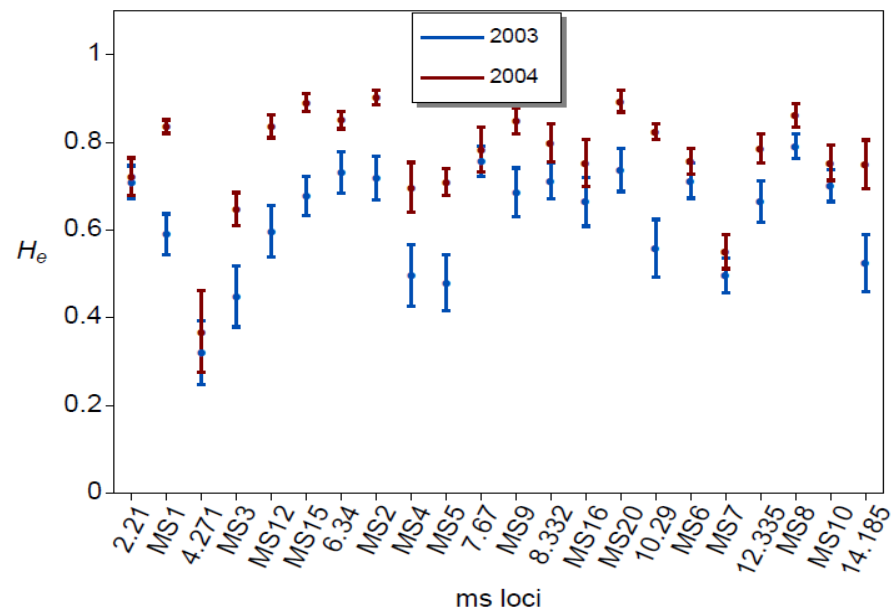

Supplement: Additional file 1 — Number of alleles per loci and expected heterozygosity per year in P. vivax (A and B) and in P. falciparum (C and D) using all samples from Tumeremo. [file 1475-2875-11-412-S1.pdf]
